# An Uncommon Case of Primary Leptomeningeal Melanoma in a 66-Year-Old White Caucasian Male

**DOI:** 10.7759/cureus.10793

**Published:** 2020-10-04

**Authors:** Ashutosh Mohapatra, Priyam Choudhury

**Affiliations:** 1 Department of Orthopaedics, Mohapatra Fracture and Accident Hospital, Mumbai, IND; 2 Department of Epidemiology and Public Health, Dr. D.Y. Patil Medical College and Hospital, Pune, IND

**Keywords:** primary leptomeningeal melanoma, mri, cns, immunohistochemistry

## Abstract

Primary melanocytic tumors of the central nervous system (CNS) arise from leptomeningeal melanocytes and possess a variable degree of aggressiveness. They have been classified into melanomatosis, melanocytoma, malignant melanoma, and diffuse melanocytosis. Melanocytic lesions of the CNS include both benign (leptomeningeal melanocytosis, melanocytoma) and malignant (leptomeningeal melanomatosis, melanoma) pathologies and the extent of anatomical site involvement dictates their clinical features. Primary CNS melanoma accounts for approximately 1% of all melanoma cases with a peak incidence in the fourth decade. Though the most common location of occurrence is in the lumbar region, our patient presented with a thoracolumbar lesion. We present a case of a 66-year-old white Caucasian male who presented with complaints of headache for six months and was thoroughly evaluated leading to a rare diagnosis of primary leptomeningeal melanoma.

## Introduction

Primary melanocytic tumors of the central nervous system (CNS) arise from leptomeningeal melanocytes and possess a variable degree of aggressiveness [1]. The World Health Organization classified these lesions into melanomatosis, melanocytoma, malignant melanoma, and diffuse melanocytosis [1,2]. Melanocytic lesions of the CNS include both benign (leptomeningeal melanocytosis, melanocytoma) and malignant (leptomeningeal melanomatosis, melanoma) pathologies and the extent of anatomical site involvement dictates their clinical features [3,4]. Primary leptomeningeal melanomatosis has a poor prognosis with the malignant melanocytes invading the Virchow-Robin spaces [5]. Meningeal melanocytoma occurs mostly in the extramedullary intradural compartment of cervical and thoracic spine with a predilection for the fifth decade and female population [6,7]. Primary CNS melanoma accounts for approximately 1% of all melanoma cases with a peak incidence in the fourth decade [8]. There is still some uncertainty about the cases of primary melanoma which in retrospect could have been secondary. Symptoms seen are intracranial hypertension, hydrocephalus, subarachnoid haemorrhage, focal neural deficits, and seizures. They are seen either as isolated cases or in conjunction with neurocutaneous melanosis [3].

## Case presentation

**Figure 1:**
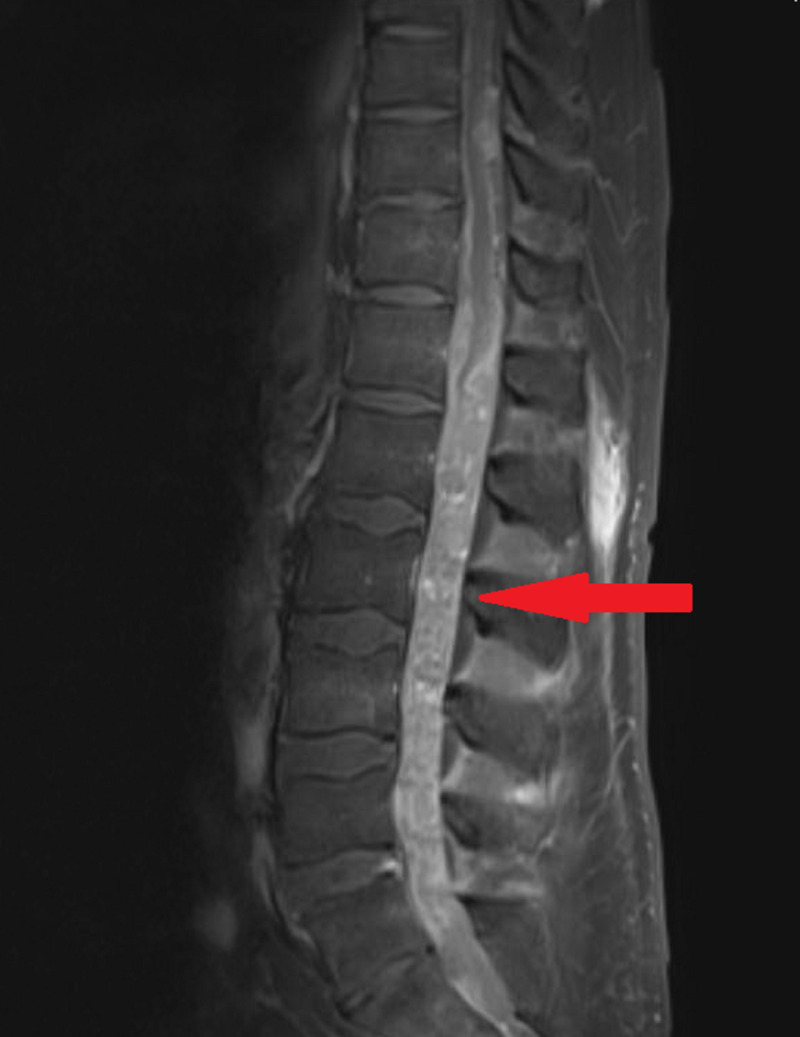
MRI spine (T1-sagittal) showing leptomeningeal enhancement along the lower thoracic and lumbar cord (red arrow)

**Figure 2:**
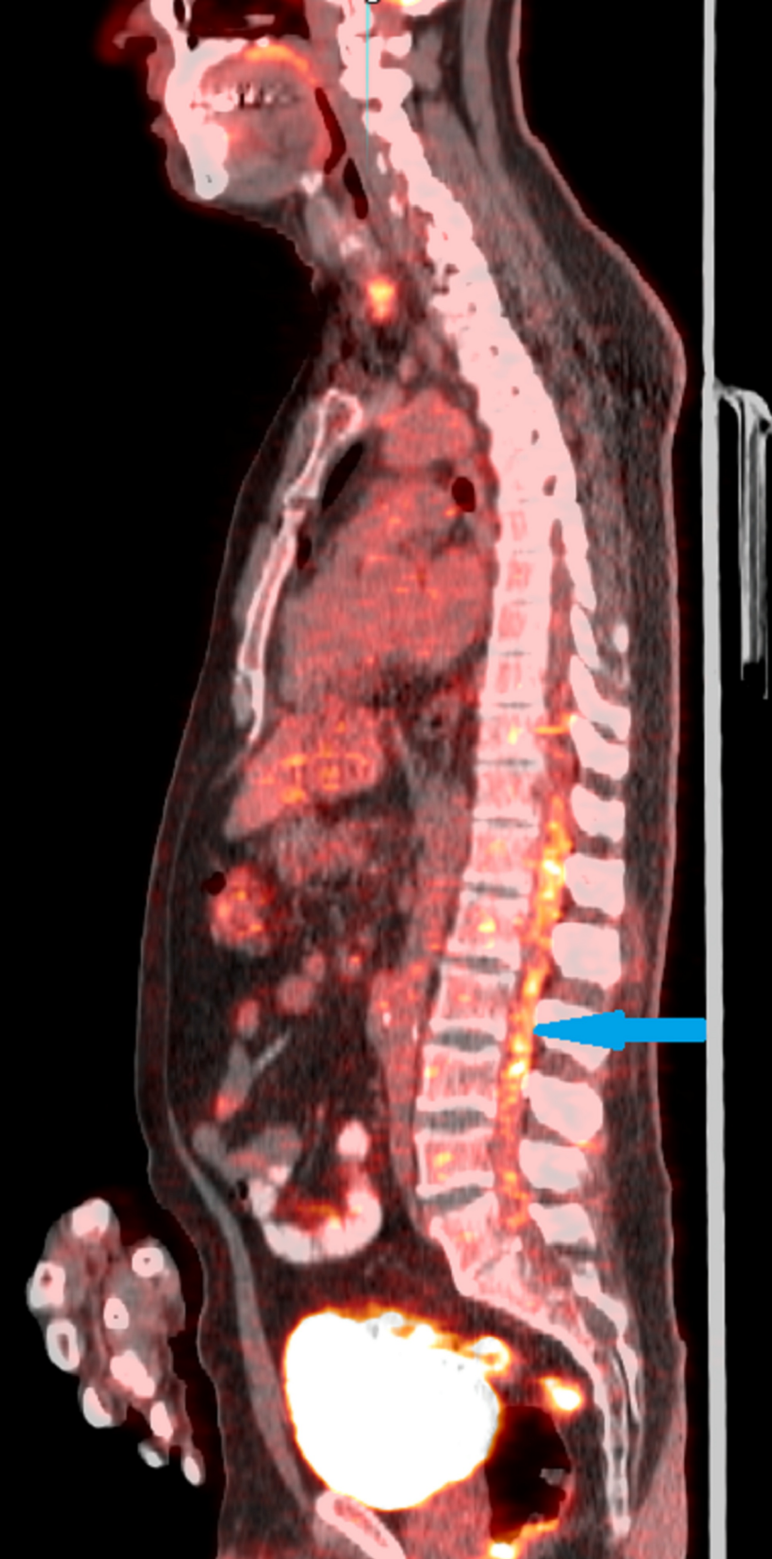
Positron emission tomography-computed tomography (PET-CT) image showing enhancement at the thoracolumbar region (blue arrow)

A 66-year-old white Caucasian male, presented to the outpatient department (OPD) with complaints of headache since two years. The headaches were mild and intermittent in nature for which he did not seek any medical intervention but took over-the-counter (OTC) medications. On the day of presenting to us, he complained of severe headache with blurring of vision. He was evaluated by a neuro-opthalmologist and was noted to have venous engorgement and loss of venous pulsation along with haemorrhages on the optic disc. No other neurovascular deficit was seen. Since these findings were of papilledema, he underwent a total of three therapeutic spinal taps to decrease the raised intracranial pressure which led to an improvement in his symptoms. The cerebrospinal fluid (CSF) sample was sent for evaluation and yielded a negative result for malignancy. Magnetic resonance imaging (MRI) brain was inconclusive too. Three months after his complaints of headache, the patient complained of lower back pain, heaviness in his lower extremities along with occasional shooting pains down his bilateral lower limbs. Further questioning revealed bowel incontinence. Clinically there was severe tenderness in the thoraco-lumbar region from T10 - L5 with paraesthesia. He had a limping gait due to pain-visual analog scale (VAS): 6 with power grade 3. He then underwent an MRI of the spine which revealed leptomeningeal enhancement from levels T10 to L4 (Figure 1). A positron emission tomography-computed tomography (PET-CT) was performed which also revealed enhancement at the same levels (Figure 2).

The patient underwent an L2 laminectomy and with an intradural biopsy of the tumour. Complete excision of the tumour was not possible. The biopsy sample was sent for histopathological evaluation which revealed densely cellular neoplasm comprising of moderately pleomorphic bean shaped to spindled tumour cells with numerous nuclear grooves and small eosinophilic nucleoli. The tumour showed a solid growth pattern with extensive infiltration and encasing of peripheral nerves. The mitotic index was 2 mitoses per high-power fields (HPF) with mild focal amounts of melanin pigment and melanophages noted. Necrosis was unidentified. Immunohistochemistry performed showed a positive result for S-100 (focal), human melanoma black (HMB)‐45 (diffuse), and Melan A (diffuse). ki-67 proliferation index was elevated to 7%. The above immunophenotype, taken together with the invasive growth pattern, degree of cytologic atypia, and increased proliferative index were most consistent with the diagnosis of melanoma. Further evaluation done in the form of a comprehensive metabolic panel (CMP) 26 mutational analysis for assessment of BRAF mutation/GNAQ/GNA11 mutation revealed mutation at GNAQ exon 5 and GNA11 exon 5. Based on the above findings, a diagnosis of primary leptomeningeal melanoma was made. A three month follow up revealed weight loss of 10 kgs with significant improvement in VAS from 6 to 3. Power had improved to grade 4. He is able to walk properly, do few daily activities, control his bowels but has been constipated. He may need colonoscopy on future follow ups since bowel lesions are not uncommon. He still occasionally gets headaches on the left side of his face from the left eye to the ear canal.

## Discussion

Primary CNS melanoma is a rarity constituting about 1% of all cases and 0.07% of the brain tumours [9]. Though the most common location of occurrence is in the lumbar region, our patient presented with a thoracolumbar lesion [10]. CT and MRI show primary diffuse leptomeningeal melamatosis (PDLM) as diffuse meningeal thickening, whereas primary leptomeningeal melanoma as dense and nodular [5]. The melanocytic lesions appear as isointense/hyperintense and hypointense on T1 and T2-weighted imaging respectively due to the paramagnetic properties of melanin [5,11]. For the confirmation of diagnosis, imaging must be supplemented with immunohistochemistry studies and histopathology since the radiographic features are non-specific [1,3]. Histologically PDLM consists of dense sheets of pleomorphic, spindle-shaped cells with melanin with features of malignancy [5,12]. On the other hand, melanocytomas are histologically cellular lesions with varying amounts of melanin pigment in their cytoplasm. Melanocytomas do not possess atypia, nuclear pleomorphism, and no more than the very occasional mitotic figure. Melanomas on the contrary are more pleomorphic, have a more anaplastic nuclei, and possess a higher cell density with tissue invasion [13]. The diagnosis of primary leptomeningeal melanoma is based on Hayward’s criteria which states the following: no malignant melanoma outside the CNS, an absence of this lesion in another area of the CNS, and histopathological confirmation [14]. Our patient fulfilled all three criteria. There are no specific guidelines for the management of both the primary CNS melanomas and leptomeningeal melanomatosis on account of their rare nature and survival being poor. However, radiotherapy especially after surgical resection of a primary tumour and systemic chemotherapy with agents such as cisplatin, dacarbazine, or thalidomide or temozolomide or immunmodulatory agents such as peginterferon alpha-2b have all been used as treatments [15]. The treatment of choice in melanomas is surgical excision of the tumour, but our patient had a dense and extensive infiltrating tumour, hence, surgery was opted out and he was started on chemotherapy.

## Conclusions

Primary leptomeningeal melanoma is a rare tumour constituting only 0.07% of all brain tumours whose diagnosis is based on Hayward’s criteria. The extent of the tumour spread dictates the clinical features seen. Due to the non specific nature of the radiographic features, imaging must be supplemented with immunohistochemistry studies and histopathology for the confirmation of diagnosis. Since they are rare in nature with a poor survival rate, their management consists of resection of primary tumour in possible cases. Due to the high radioresistant nature of the tumour, systematic treatment is preferred in most of the cases.
